# A Scleroderma and Raynaud’s UK (SRUK) national survey to explore rheumatologists’ awareness, approaches to diagnosis and management and training needs within systemic sclerosis

**DOI:** 10.1093/rap/rkae152

**Published:** 2024-12-20

**Authors:** Emma Blamont, Sue Farrington, Nina Glover-Southworth, Gemma Cornwell, Laura Gibson, Melanie Sloan, Michael Hughes

**Affiliations:** Scleroderma and Raynaud’s UK, London, UK; Scleroderma and Raynaud’s UK, London, UK; Scleroderma and Raynaud’s UK, London, UK; Scleroderma and Raynaud’s UK, London, UK; Scleroderma and Raynaud’s UK, London, UK; Department of Public Health and Primary Care, University of Cambridge, Cambridge, UK; Division of Musculoskeletal and Dermatological Sciences, The University of Manchester, Manchester Academic Health Science Centre, Manchester, UK; Department of Rheumatology, Northern Care Alliance NHS Foundation Trust, Salford Care Organisation, Salford, UK

**Keywords:** scleroderma, systemic sclerosis, diagnosis, management, follow-up, training, education

## Abstract

**Objectives:**

Patients’ outcomes and experiences can be affected by rheumatologist knowledge and awareness of systemic sclerosis (SSc). Our survey, directed at UK-based rheumatologists, aimed to expand our understanding of the above points along with their ability to keep up to date with guidelines defining best practice.

**Methods:**

Rheumatologists were invited to participate in an online survey, with the results analysed and presented descriptively and graphically by SRUK.

**Results:**

Of the 150 UK rheumatologists surveyed, 2% reported that they had not heard of SSC and 18.7% reported having a limited understanding of the condition. A total of 44% of respondents reported that they were fully versed in the signs and symptoms of SSc. The majority of those surveyed shared the broad view that all aspects of SSc, including services (63.4%), specialist positions (64.7%), research (73.1%) and training and education (70%), are either completely or somewhat underfunded. Most respondents (87.4%) reported that their workload allowed them to ‘somewhat’ (48.74%) or ‘completely’ (38.66%) keep up to date with official guidelines. Scleroderma and Raynaud’s UK (SRUK) (47.9%), followed by NICE (43.7%), were the most highly used sources of information utilized among those surveyed.

**Conclusion:**

Our survey reveals a serious gap in the awareness and the signs and symptoms of SSc among some UK rheumatologists, in addition to a perception that services, training and education in this area are underfunded. Our findings indicate that there is a role for the provision of further education and training as part of continued professional development.

Key messages

- Around 2% of surveyed UK rheumatologists had not heard of SSc and ≈19% did not fully understand its bodily impact.
- More than half of those responding believed that SSc research and services are underfunded.
- The majority (≈90%) of those surveyed would like to receive further training in SSc.

## Introduction

SSc, often referred to as ‘scleroderma’, is a rare autoimmune rheumatic disease affecting an estimated 19 000 of the UK population and ≈2.5 million globally [1–4]. It is a complex and heterogeneous condition affecting the skin and internal organs such as the lungs, gastrointestinal tract, heart and kidneys [1, 3]. Progressive systemic vasculopathy, aberrant tissue fibrosis and immune system activation results in the progressive accumulation of tissue damage and organ dysfunction [1, 5].

SSc has the highest mortality of any of the autoimmune rheumatic diseases. Around half of those affected will eventually die from the disease of SSc-related complications [1, 6, 7]. Diagnostic delay is an important problem within SSc [4] and can directly impact disease outcome due to the accumulation of widespread tissue damage and organ dysfunction. The systemic nature of the condition means that patient management is highly complex, with many patients requiring care in addition to rheumatology services from an array of medical and allied health specialties [8, 9].

Patient experience and outcomes can thus be influenced by the knowledge and awareness of SSc among rheumatologists and other healthcare specialists and the closeness with which these teams work together. To better understand these factors, Scleroderma and Raynaud’s UK (SRUK) undertook a national survey to understand rheumatologists’ knowledge and awareness of SSc, their approach to managing the condition in their patients and their needs for further education and training.

## Methods

### Study design

The study was commissioned by SRUK and designed by the market research agency CENSUSWIDE following a brief from the survey Steering Board comprising in-house expertise from SRUK and a rheumatologist with a specialist interest in SSc (M.H.). The resulting group co-produced a bespoke survey to explore rheumatologists’ awareness and knowledge of SSc, their approaches to patient management and follow-up and their needs for further training and education in SSc.

### Information surveyed

The survey consisted of a series of questions (see Supplementary Data S1, available at *Rheumatology Advances in Practice* online) that included basic clinician-reported demographics, years of clinical experience and their experience with SSc (knowledge/understanding of SSc and awareness of its signs and symptoms, specialist interest, how many SSc patients under their care). To better understand approaches to diagnosis and care, rheumatologists were asked about the frequency of patient follow-up, the frequency at which they administer assessments and tests and the diagnostic capabilities within their National Health Service (NHS) trust (autoantibody testing and nailfold capillaroscopy). Information usage by clinicians was explored by asking which information sources are used, such as those produced by patient organizations [SRUK and the Rare Autoimmune Rheumatic Disease Alliance (RAIRDA)], public bodies [NHS England and the National Institute for Health and Clinical Excellence (NICE)] and professional societies [British Society for Rheumatology (BSR)], and whether their role allows them to easily keep up to date with best practice guidance.

### Study participants and inclusion criteria

The survey was completed by 150 rheumatologists identified through CENSUSWIDE and their responses were collated. Respondents were screened for eligibility through a series of triaging questions. To participate, respondents had to be at least 23 years of age and be in full- or part-time employment as a medical doctor (junior doctor through to consultant/clinical academic grade) specializing in rheumatology. Participants who reported that they lacked any knowledge or understanding of SSc were removed from our present analysis since these questions focused on the experiences, treatment and care of patients with SSc.

### Ethics and consent

Ethical approval was deemed unnecessary for our study because no personal information was collected throughout, participation was voluntary and respondents could terminate their participation at any point. The Health Research Authority decision tool (www.hra-decisiontools.org.uk/research/) confirmed that the present study would not be considered as ‘research’ by the UK Policy Framework for Health and Social Care Research. Respondents were recruited online as opposed to through the NHS. Through the action of completing the survey the respondents gave their agreement to the use of their anonymous answers to address the study objectives.

### Statistical analyses

Data were imported from the survey platform into Excel (Microsoft, Redmond, WA, USA) and SPSS (IBM, Armonk, NY, USA). Descriptive statistics were used to summarize the data. The absolute and relative frequencies were calculated and depicted in tabular and graphical form. Data are presented as the number and percentage of all available responses to each individual question throughout the article. We investigated whether the difference in mean values between different groups of clinicians and different responses (e.g. self-reported level of knowledge of SSc) were statistically significant using *t*-tests. An α <0.05 was used as the cut-off for significance.

## Results

### Clinician demographics and experience of SSc

**Figure 1.**
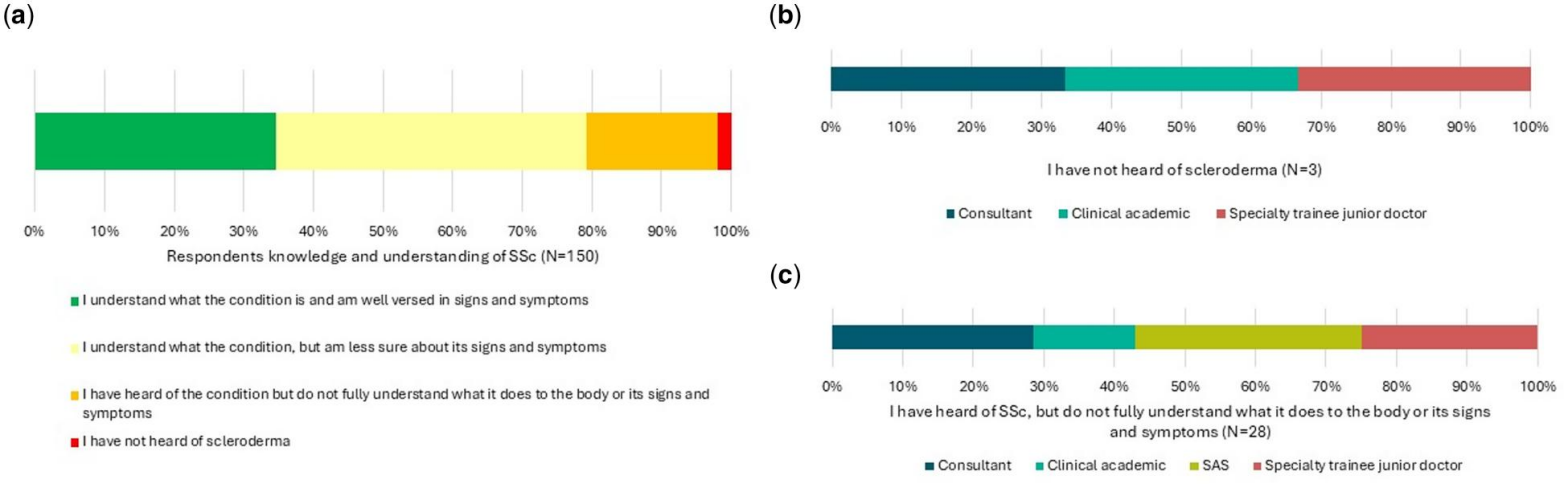
Respondents’ knowledge and understanding of SSc and its signs and symptoms. **(A)** Respondents (*N* = 150) were asked to select which of the four knowledge categories indicated in the legend best matched their level of knowledge and understanding. Percentages of respondents selecting each category are indicated on the *x*-axis. **(B)** Clinical grade of respondents (*n* = 3) self-reporting that they had never heard of SSc. **(C)** Clinical grade of respondents (*n* = 28) self-reporting that they had limited understanding of the effects of SSc on the body or its signs and symptoms

The survey was targeted towards 150 UK-based rheumatologists. Thirty-one respondents had either not heard of SSc [*n* = 3 (2%)] or reported a lack of understanding of the condition along with its signs and symptoms [*n* = 28 (18.7%)], leaving 119 evaluable responses (Fig. 1, Table 1). The majority of those responding identified as being non-SSc specialists [*n* = 77 (64.7%)]. There was wide participation from around the UK and representation from all career stages from specialty trainee clinical doctors through to consultant and clinical academics. Years of experience varied, with a mode of 6–10 years of experience [*n* = 50 (42%)]. A correlation analysis showed no correlation between years of practice and knowledge of SSc (*P* = 0.8).

**Table 1.** Characteristics of the survey respondents (*N* = 119)

	Clinician characteristics	*n* (%)
Age (years)	23–34	17 (14.29)
	35–44	49 (41.18)
	45–54	38 (31.93)
	≥55	15 (12.61)
Gender	Male/female	86 (72.27)/32(26.89)
	Prefer not to say	1 (0.84)
Region	Greater London	44 (36.97)
	South East	16 (13.45)
	East of England	10 (8.40)
	Midlands	9 (7.56)
	North East	5 (4.20)
	North West	5 (4.20)
	South West	4 (3.36)
	Yorkshire and Humber	1 (0.84)
	Scotland	16 (13.45)
	Wales	6 (5.04)
	Northern Ireland	3 (2.52)
Years experience	<1	6 (5.04)
	1–5	26 (21.85)
	6–10	50 (42.02)
	11–15	28 (23.53)
	16–20	7 (5.88)
	≥21	2 (1.68)
Level	Clinical academic	18 (15.13)
	Consultant	27 (22.69)
	SAS (specialist, associate specialist or specialty)	52 (43.70)
	Specialty trainee junior doctor	22 (18.49)
SSc specialist	Yes	42 (35.29)
	No	77 (64.71)
Number of SSc patients currently seen	<5	10 (8.40)
	1–25	23 (19.33)
	26–55	18 (15.13)
	56–99	14 (11.76)
	100–199	13 (10.92)
	200–299	16 (13.45)
	300–399	16 (13.45)
	400–499	4 (3.36)
	500–599	4 (3.36)
	1000–2500	1 (0.84)

### Awareness of SSc and diagnostic capabilities

Of the 119 evaluable responses from respondents reporting an understanding of SSc, just 44% (*n* = 52) reported that ‘they were well versed in the signs and symptoms of scleroderma’, while the remainder [*n* = 67 (56%)] stated they were ‘not fully aware of all signs and symptoms’. When questioned, most respondents said that their NHS trust had the necessary equipment and staff capabilities to inform a diagnosis of SSc through screening for autoantibodies [Yes, *n* = 101 (84.9%)] or use of nailfold capillaroscopy to diagnose scleroderma [Yes, *n* = 100 (84%)].

### Management of SSc and perceived adequacy of treatment options

**Figure 2.**
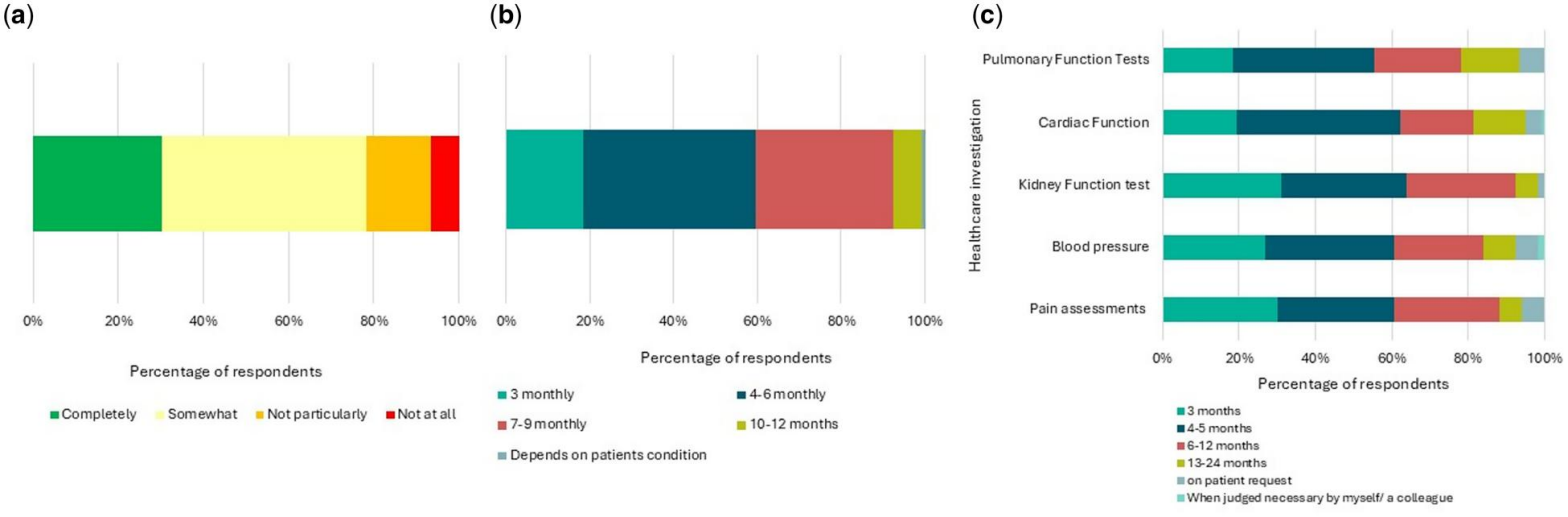
Treatment and management of SSc. Responses were collated from respondents within knowledge and understanding of SSc (*N* = 119). **(A)** Perceived adequacy of treatment options for SSc as reported by survey respondents. Percentages of responses against each adequacy category are indicated on the *x*-axis. **(B)** Frequency at which SSc patients are reviewed in months. Percentage of respondents performing reviews from the evaluable responses are indicated on the *x*-axis. The frequency of review is indicated in the legend. **(C)** Frequency at which health/organ function assessments are performed by respondents on their SSc patients. Tests or investigations are indicated on the *y*-axis, the percentage of respondents (*N* = 119) carrying out the assessment is shown on the *x*-axis and the frequency of assessment is indicated in the legend

The number of patients currently seen with the condition varied greatly among those surveyed. Most respondents had 5–25 SSc patients [*n* = 23 (19.3%)], however, some reported having <5 patients [*n* = 10 (8.4%)] or many more, with one clinician reporting having 1000–2500 SSc patients [*n* = 1 (0.8%)] under his/her care. Current available treatment options for scleroderma were deemed to be ‘completely’ or ‘somewhat’ adequate [*n* = 36 (30.3%) or *n* = 57 (47.9%), respectively]. Those self-identifying as SSc specialists had a higher mean rating for perceiving that treatment was adequate (*P* < 0.001) (Fig. 2A).

Frequency of patient follow-up was variable. Most clinicians stated that they review patients every 4–6 months [*n* = 49 (41.2%)] or every 7–9 months [*n* = 39 (32.8%)], however, some reported that they see their patients more frequently, at every 3 months [*n* = 22 (18.5%)]. Only 6.7% (*n* = 8) of respondents reported seeing their patients on an annual basis of every 10–12 months (Fig. 2B). Just one clinician [*n* = 1 (0.8%)] he/she saw patients based upon the patient’s condition.

Most respondents routinely perform tests for pulmonary function [*n* = 111 (93.3%)], cardiac function [*n* = 113 (95%)], kidney function [*n* = 117 (98.3%)], blood pressure [*n* = 110 (92.4%)] and pain assessment [*n* = 112 (94.1%)] as part of their patient care. There were varied responses regarding the frequency of testing depending on the test used, however, respondents most commonly reported using these assessments on a 4- to 5-month basis {pulmonary function [*n* = 44 (37%)], cardiac function [*n* = 51 (42.9%)], kidney function [*n* = 39 (32.8%)], blood pressure [*n* = 40 (33.6%)] and pain assessment [*n* = 26 (30.3%)]}. Some clinicians reported not using these tests routinely, instead stating that they are given when requested by a patient or when deemed necessary by themselves or a colleague {pulmonary function [*n* = 8 (6.7%)], cardiac function [*n* = 6 (5%)], kidney function [*n* = 2 (1.7%)], blood pressure [*n* = 9 (7.6%)] and pain assessment [*n* = 7 (5.9%)]} (Fig. 2C).

### Multidisciplinary working

**Figure 3.**
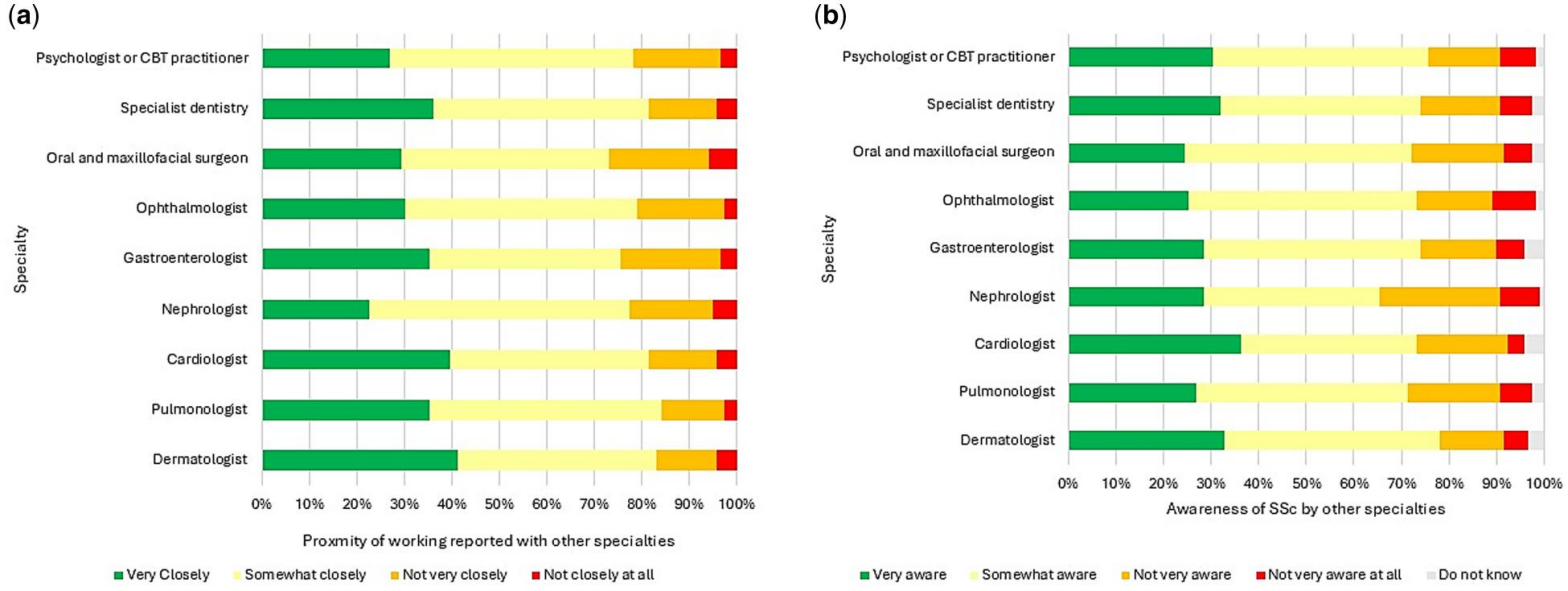
Involvement of other specialists in the care of SSc patients along with specialist awareness of SSc as a condition as reported by those surveyed. Responses are taken from the 119 evaluable responses with knowledge and understanding of SSc. **(A)** Proximity between rheumatology with other specialists in the management of SSc patients. Specialists are indicated on the *y*-axis, proximity categories are within the legend and the percentage of rheumatology respondents reporting against each category is indicated on the *x*-axis. **(B)** Awareness of SSc among specialists from fields likely to be involved in the care of SSc as quoted by those surveyed. Specialists are indicated on the *y*-axis, awareness categories are indicated in the legend and the percentage of rheumatology respondents reporting against each awareness category is on the *x*-axis

Given the complexity and heterogeneity of SSc, we were keen to explore how closely the respondents worked with other specialists involved in patient care and the general awareness these specialists had of the condition. Most of those surveyed responded that they worked either closely or somewhat closely with specialist colleagues in pulmonology [*n* = 100 (84%)], dermatology [*n* = 99 (83.2%)], cardiology [*n* = 97 (81.5%)], specialist dentistry [*n* = 97 (81.5%)], ophthalmology [*n* = 94 (79%)], psychology/cognitive behavioural therapy (CBT) [*n* = 93 (78.1%)], nephrology [*n* = 92 (77.3%)], gastroenterology [*n* = 90 (75.6%)] and oral and maxillofacial surgery [*n* = 87 (73.1%)] (Fig. 3A). These specialists had an awareness of SSc, with most respondents quoting that each of these colleagues were ‘somewhat or very aware’ {dermatology [*n* = 93 (78.2%)], pulmonology [*n* = 85 (71.4%)], cardiology [*n* = 87 (73.1%)], nephrology [*n* = 78 (65.6%)], gastroenterology [*n* = 88 (74%)], ophthalmology [*n* = 87 (73.1%)], oral and maxillofacial surgery [*n* = 86 (72.3%)], specialist dentistry [*n* = 88 (74%)] and psychology/CBT [*n* = 90 (75.6%)]} (Fig. 3B). Compared with non-SSc specialists, those self-identifying as SSc specialists rated other specialties knowledge of SSc as higher. This was significant for cardiologists (*P* = 0.041), gastroenterologists (*P* = 0.03) and ophthalmologists (*P* = 0.041).

### Perceived adequacy of funding for SSc compared with other conditions

**Figure 4.**
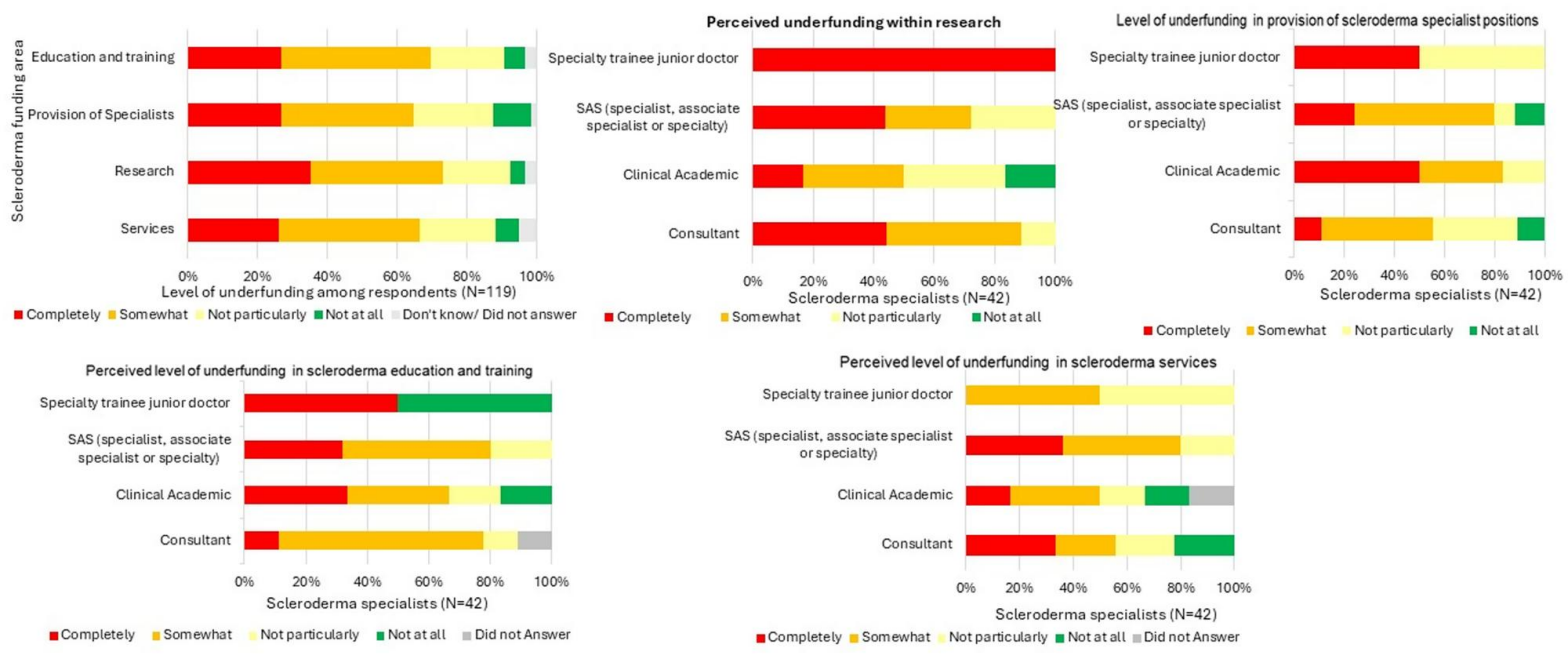
Perception of underfunding within SSc as reported by survey respondents. **(A)** Perception of underfunding within SSc as reported from evaluable survey responses (*N* = 119). Perceived level of underfunding within SSc education and training, specialist provisions, research and services as reported by UK rheumatologists. Areas of potential funding need are indicated on the *y*-axis and the extent to which they are perceived to be underfunded by rheumatologists, expressed as a percentage, is reported on the *x*-axis. Perception of underfunding within **(B)** SSc research, **(C)** provision of SSc specialist positions, **(D)** education and training and **(E)** SSc services as reported by respondents self-identifying as SSc specialists (*n* = 42). Clinical grades are indicated on the *y*-axis and the extent to which they are perceived to be underfunded, expressed as a percentage, is reported on the *x*-axis

The level of funding available for SSc can directly impact patient care and experience. We wished to explore perceptions among respondents into the funding available for SSc services, research into better diagnosis and treatments, SSc specialist positions and training and education of healthcare professionals. Most respondents thought that resources were somewhat or completely underfunded compared with other conditions. This included research [*n* = 87 (73.11%)], training and education [*n* = 83 (69.75%)], specialist positions [*n* = 77 (64.71%)] and services [*n* = 79 (63.4%)] (Fig. 4A). We were interested to explore if the perceived lack of funding within the above areas reflects the actual situation or may be influenced by the rarity of the condition and the bias towards responses from non-SSc specialists. To better understand this, we analysed the responses from those identifying as SSc specialists (*n* = 42) (Fig. 4B–E). More than 70% of ‘scleroderma specialists’ responded that each of these areas were either completely or somewhat underfunded compared with other conditions {research [*n* = 31 (73.81%)], specialist services [*n* = 29 (70.73%)], specialist positions [*n* = 31 (73.81%)] and education and training [*n* = 32 (78.05%)]}.

### Clinician education and ability to keep up with best practice

We wanted to understand the types of information resources clinicians use to inform SSc patient care. Usage varied greatly between the rheumatologists questioned. The most turned to source was SRUK [*n* = 57 (47.9%)], followed by NICE [*n* = 52 (43.7%)], RAIRDA [*n* = 51 (42.9%)] and NHS England [*n* = 47 (39.5%)]. BSR was the least used [*n* = 45 (37.8%)].

We next wanted to establish whether workload impacts on clinician’s ability to keep up to date with official guidance/best practice published by NICE, NHS England, BSR, EULAR and other professional societies. Most rheumatologists responded that they were able to ‘somewhat’ [*n* = 58 (48.7%)] or ‘completely’ [*n* = 46 (38.7%)] easily keep up to date with guidance. Some respondents reported that their workload did not particularly allow them to easily keep up to date [*n* = 12 (10.1%)], and a very small number responded that that their workload did not allow them to easily keep up to date at all [*n* = 3 (2.5%)]. Comparing those self-identifying as SSc specialists with those who did not, SSc specialists were more likely to be able to more easily to stay up to date (*P* = 0.043).

**Figure 5.**
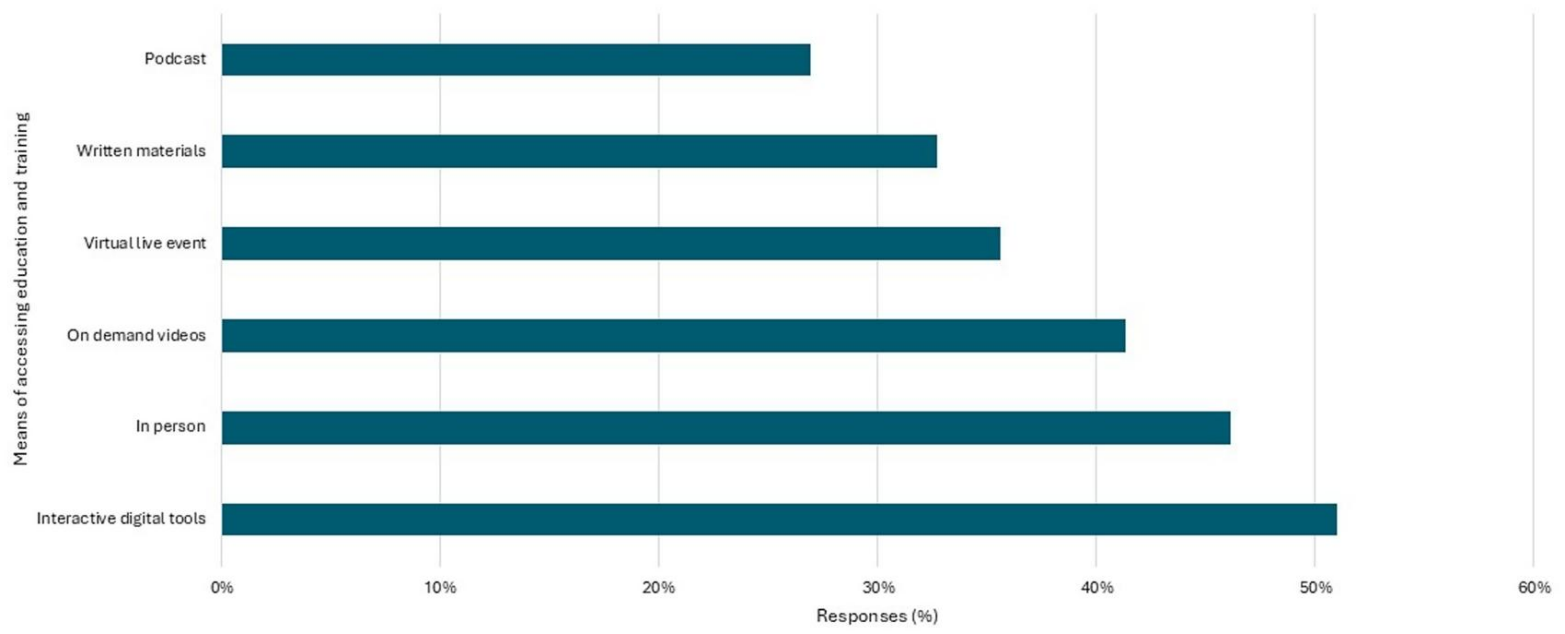
Preferences for delivery of further training and education in SSc. Respondents responding yes they would value further training and education (*N* = 104) in SSc were asked to select how they would like to access these. Options for assessing education and training are shown on the *y*-axis and the percentage of respondents (*N* = 104) selecting each option is indicated on the *x*-axis

Most rheumatologists surveyed said that they would value further training in SSc care [*n* = 104 (87.4%)]. Surprisingly, there were no significant differences between SSc specialists and non-specialists, age and years of practice in the responses to this question. When asked how they would like to receive this training (Fig. 5), most opted for training via interactive digital tools [*n* = 53 (51%)] followed by in-person training [*n* = 48 (46.2%)], on-demand access to video materials [*n* = 43 (41.4%)], virtual live events [*n* = 37 (35.6%)] and written materials [*n* = 34 (32.7%)]. The least popular option for receiving further training was podcasts [*n* = 28 (26.9%)].

## Discussion

To our knowledge this is the first survey benchmarking the UK rheumatology workforce’s knowledge and experience of SSc and their current practice in routine patient clinical management. A key finding of our study is that only one-third of those surveyed self-assessed as being ‘well versed in the signs and symptoms of scleroderma’, and more than one-fifth of the initial 150 respondents had never heard of SSc or had limited understanding of the condition and its signs and symptoms.

The lack of knowledge and understanding of SSc highlighted above has implications for patient diagnosis, care and long-term outcomes, especially given that diagnostic delay is commonly observed within SSc and other rare rheumatic diseases [4]. Algorithms incorporating red flags and signs concomitant with Very Early Diagnosis of Systemic Sclerosis (VEDOSS) [10] could likely help support earlier diagnosis and breach gaps in awareness, especially given most respondents indicated high levels of in-trust capabilities for performing nailfold capillaroscopy and extended SSc autoantibody testing. Furthermore, patient-led organizations also have a major role in raising awareness of the condition in the general population, including through digital-based interventions (e.g. the online SRUK Raynaud’s test) [11–13].

Our findings indicate that expert and international guidelines are usually being adhered to, with most patients being followed up every 3–9 months, and routinely tested for organ involvement/progression of organ involvement [8, 9]. Multidisciplinary working practices appear to be in place among the evaluable respondents, with most of these individuals reporting that their cross-disciplinary colleagues have an awareness of SSc.

National and international best practice guidelines are in place for SSc, such as the BSR and EULAR guidelines [8, 9, 14], which largely focus on treatments. These are revised and updated on a semi-regular basis to incorporate the rapid advances in treatment and care, such as the advent of the use of anti-fibrotic medications such as nintedanib in the treatment of progressive interstitial lung disease [15]. Keeping up with these evolving guidelines is of utmost importance given the rapid pace in changes to early diagnosis (e.g. VEDOSS) and the ever-decreasing diagnostic (threshold) criteria for pulmonary hypertension [16]. Indeed, most rheumatologists surveyed indicated that their workload allowed them to keep abreast of best practices and official guidelines to some extent. Again, there is clearly a central role for patient-led organizations to play in disseminating official guidance to patients and clinicians alike. For example, SRUK was the resource most frequently used by the clinicians surveyed to inform their direct patient care.

Our results also indicate a perception among rheumatologists that SSc is underfunded compared with other diseases. Most rheumatologists who participated highlighted a need for greater funding of research, services and clinical posts. Education was also a need area explored within our survey, with most respondents (87.4%) stating that they would value further training in SSc and indicating a preference for interactive on-line digital training materials [*n* = 53 (51%)]. This warrants the development of a cross-agency training platform.

A significant strength of our study was that the selection biases in the survey inclusion have been minimized due to the recruitment methods used by CENSUSWIDE. However, our study has a number of important limitations, including the sample size. Self-reported data are also subject to several potential biases, including social desirability bias. Our results are skewed towards the views and practices of those with more knowledge/experience due to the exclusion of participants, with more than one-fifth of those responding that they had never heard of SSc or had limited understanding of the condition and its signs and symptoms. It should be highlighted that there was a significant proportion of SAS-level (compared with consultant) senior medical clinicians who completed the survey. The reasons for this are unclear and could include (but are not limited to) a selection bias to whose respondents who were engaged and motivated to participate in the survey. Nonetheless, no significant differences (data not shown here) were observed in the responses between these senior-level medical rheumatology clinicians. Indeed, these data highlight the importance of supporting (including through further dedicated continuing professional education opportunities) these important senior members of the rheumatology medical workforce. Another aspect is that there was no formal pre-agreed or pre-published protocol/statistical analysis plan, although our analysis was mainly descriptive, and analysis between groups was conducted using simple statistical testing and with a standard level of accepted statistical significance. Another important consideration is that (UK) SSc research (and care) is often concentrated/coordinated in tertiary centres for the condition and may lead to greater exposure to clinicians (including those in training) working in these institutions. Future research could examine the differences between types of institutions, including experience accrued during specialist rheumatology training.

In conclusion, our survey data highlight the variable level of knowledge of SSc and management practices among UK-based rheumatologists. It reveals a gap in both knowledge and awareness among some rheumatologists and an appetite and need for greater training within this area as part of continued professional development. The high utilization of SRUK resources among those surveyed suggests a more collaborative role for patient-led organizations alongside professional bodies in helping deliver this aim.

## Supplementary Material

rkae152_Supplementary_Data

## Data Availability

The data underlying this article will be shared upon reasonable request to the corresponding author.
