# Supplementary material for: A Scleroderma and Raynaud’s UK (SRUK) national survey to explore rheumatologists’ awareness, approaches to diagnosis and management and training needs within systemic sclerosis
Source: Rheumatol Adv Pract. 2024 Dec 20;9(1):rkae152. doi: 10.1093/rap/rkae152 (PMC11739612; doi:10.1093/rap/rkae152)
Supplement: rkae152_Supplementary_Data [file rkae152_supplementary_data.zip › e81eb_24-173 Supplementary Material.docx]

**Supplementary Data S1 – SRUK Rheumatologist Survey Questions**

Qa. [Exact age]

What age group are you?

*End if 16-22 is selected*

23-34

35-44

45-54

55+

Qb. Which one of the following best describes your role?

Work full time

Work part time

Retired [End]

Home-maker / full time parent [End]

Student [End]

Unemployed [End]

Other [End]

Qc. In which one of the following industry sectors do you work?

Architecture, Engineering & Building

Arts & Culture

Education

Finance

Healthcare

HR

IT & Telecoms

Legal

Manufacturing & Utilities

Retail, Catering & Leisure

Sales, Media & Marketing

Travel & Transport

Other

[End if ‘Healthcare’ is not selected]

Qd. Which of the following best describes your area of healthcare?

Ambulance services

Dentistry

Healthcare administration and management

Healthcare science (e.g. clinical engineering, biomedical science, and pathology)

Medical equipment sales

Medicine (e.g. doctors, surgeons, and GPs)

Medical research

Midwifery

Nursing

Nutrition and diet

Optometry and opticians

Pharmacy

Psychological therapies

None of the above *exclusive*

[End if ‘Medicine (e.g. doctors, surgeons, and GPs)’ is not selected]

Qe. Which of the following best describes your title / main field of research?

Anaesthesiologist

Cardiologist

Dermatologist

Endocrinologist

Family medicine

Gastroenterologist

General practitioner

Internal medicine

Neurologist

Neurology

Obstetrics and gynaecology

Oncologist

Ophthalmologist

Orthopaedist

Paediatrician

Pathologist

Radiologist

Rheumatologist

Surgeon

Urologist

[End if ‘Rheumatologist’ is not selected at Qe]

1. How many years of experience do you have as a rheumatologist?

a. Less than a year

b. 1-5

c. 6-10

d. 11-15

e. 16-20

f. 21+, please specify in years

2. How aware are you of scleroderma as a medical condition, if at all?

a. I understand what the condition is and am well versed in signs and symptoms

b. I understand what the condition, but am less sure about its signs and symptoms [Skip Q3]

c. I have heard of the condition but do not fully understand what it does to the body or its signs and symptoms [Skip Q3]

d. I have not heard of scleroderma [Skip Q3]

e. Other (please specify)

3. Are you a Scleroderma specialist?

a. Yes

b. No [Assign if ‘I understand what the condition, but am less sure about its signs and symptoms’ or ‘I have heard of the condition but do not fully understand what it does to the body or its signs and symptoms’ or ‘I have not heard of scleroderma‘ is selected at Q2]

4. How many patients with scleroderma do you currently care for?

a. None [Skip Q5 and Q6]

b. Fewer than 5

c. 5-25

d. 26-55

e. 56-99

f. 100-199

g. 200-299

h. 300-399

i. 400-499

j. 500-999

k. 1,000-2,500

l. 2,501-5,000

m. More than 5,000, please specify

n. N/A - we refer all patients with scleroderma to a more specialist hospital [Skip Q5, Q6 and Q14]

5. How often do you routinely see your patients with scleroderma?

a. Every 3 months or more often

b. Every 4-6 months

c. Every 7-9 months

d. Every 10-12 months

e. Less often than once a year, please specify once every x years

f. It depends on each patient’s condition

6. How often, if ever, do you perform the following checks on your patients with scleroderma?

*Matrix*

Rows:

a. Pulmonary function tests

b. Cardiac function test

c. Kidney function test

d. Blood pressure test

e. Pain assessments

Columns:

i. Every 3 months or more often

ii. Every 4-5 months

iii. Every 6-12 months

iv. Every 13-24 months

v. Only when the patient requests it

vi. When either myself or a colleague deems it necessary

7. Does your NHS Trust have the necessary equipment and staff capabilities to run all tests required to diagnose scleroderma?

a. Yes

b. No

c. Do not know

8. Which information sources do you use to inform the care you provide to your patients with scleroderma? Tick all that apply

a. NICE

b. Scleroderma & Raynaud’s UK (SRUK)

c. British Society of Rheumatology (BSR)

d. RAIRDA

e. NHS England

f. Other (please specify)

g. None *exclusive*

h. Do not know *exclusive*

9. To what extent, if at all, do you feel that your overall workload enables you to easily stay up-to-date with and always follow guidance from NICE and NHS England for the treatment and care of people with scleroderma?

a. Completely

b. Somewhat

c. Not particularly

d. Not at all

e. N/A

f. Prefer not to say

10. To what extent, if at all, do you think current treatment options for scleroderma are adequate?

a. Completely

b. Somewhat

c. Not particularly

d. Not at all

e. N/A

f. Do not know

11. To what extent, if at all, do you think the following are underfunded compared with other medical conditions?

*Matrix*

Rows:

a. Scleroderma services

b. Research to find better ways to diagnose and treat scleroderma

c. Provision of scleroderma specialists

d. Scleroderma training and education for healthcare professionals

Columns:

i. Completely

ii. Somewhat

iii. Not particularly

iv. Not at all

v. N/A

vi. Do not know

12. Some scleroderma patients may need to be referred to other specialists (e.g. dermatologist, pulmonologist, cardiologist). How aware of scleroderma are your colleagues, if at all?

*Matrix*

Rows:

a. Dermatologist

b. Pulmonologist

c. Cardiologist

d. Nephrologist

e. Gastroenterologist

f. Ophthalmologist

g. Oral and maxillofacial surgeon

h. Specialist dentistry

i. Psychologist or CBT practitioner

Columns:

i. Very aware

ii. Somewhat aware

iii. Not very aware

iv. Not very aware at all

v. Do not know

13. How closely, if at all, do you work with other specialists (e.g. dermatologist, pulmonologist, cardiologist) when providing care to patients with scleroderma?

*Matrix*

Rows:

a. Dermatologist

b. Pulmonologist

c. Cardiologist

d. Nephrologist

e. Gastroenterologist

f. Ophthalmologist

g. Oral and maxillofacial surgeon

h. Specialist dentistry

i. Psychologist or CBT practitioner

Columns:

i. Very closely

ii. Somewhat closely

iii. Not very closely

iv. Not closely at all

14. Would you value further training in scleroderma care?

a. Yes

b. No [Skip Q15]

c. Don’t know [Skip Q15]

15. How would you like further training in scleroderma care delivered?

a. In-person training event

b. Virtual ‘live’ event

c. On demand access to video materials

d. Podcast

e. Interactive digital tools

f. Written materials only

g. Other (please specify)

h. Do not know

Qi. What gender are you?

Male

Female

Qii. Where do you live?

East of England

Greater London

East Midlands

West Midlands

North East

North West

Northern Ireland

Scotland

South East

South West

Wales

Yorkshire and the Humber

Qiii. Which one of the following cities do you live in or closest to?

Belfast

Birmingham

Brighton

Bristol

Cardiff

Edinburgh

Glasgow

Leeds

Liverpool

London

Manchester

Newcastle

Nottingham

Norwich

Plymouth

Sheffield

Southampton

Qiv. What level of doctor are you?

a. Specialty trainee junior doctor

b. SAS (specialist, associate specialist or specialty)

c. Consultant

d. Clinical academic

e. Other (please specify)
